# Problematic social media use and psychosocial conditions

**DOI:** 10.3389/fpsyg.2026.1778148

**Published:** 2026-03-27

**Authors:** Håkan Stattin, Charli Eriksson, Einar B. Thorsteinsson

**Affiliations:** 1 Uppsala University, Department of Psychology, Uppsala, Sweden; 2 Karolinska Institutet, Department of Learning, Informatics, Management and Ethics, Stockholm, Sweden; 3 University of New England, Armidale, NSW, Australia

**Keywords:** adolescents, cluster analysis, cyberbullying, digital media use, mental health indicators, person-centered analysis, problematic social media use (PSMU), psychosocial maladjustment

## Abstract

**Background:**

Problematic social media use (PSMU) is frequently associated with adolescent mental health problems, but the interpretive meaning of a high PSMU score in population surveys remains unclear. We examined whether elevated PSMU identifies adolescents with broader psychosocial difficulties or appears in multiple psychosocial contexts.

**Methods:**

Cluster analysis was conducted using data from the 2022 Health Behaviour in School-aged Children (HBSC) survey, including nationally representative samples of 15-year-old adolescents (mean age 15.80 years, SD 0.41) from five Nordic countries (*N* = 7,202).

**Results:**

Adolescents with high PSMU, psychosomatic complaints, and perceived stress showed the most adverse psychosocial profile, including cyberbullying involvement, negative school and peer experiences, loneliness, and low well-being. Adolescents with high psychosomatic complaints and perceived stress but average PSMU showed equal or greater difficulties, except for cyberbullying. A third group reported high PSMU without elevated perceived stress or psychosomatic complaints and did not show elevated psychosocial difficulties on the measured indicators.

**Conclusion:**

High PSMU scores occurred in heterogeneous psychosocial contexts. Adolescents with high PSMU were distributed across markedly different psychosocial profiles, and those with the most adverse stress-related conditions were often identified without elevated PSMU. These findings suggest that PSMU should be interpreted cautiously when used as a stand-alone indicator in adolescent population surveys and that its meaning depends on accompanying stress-related symptoms and social context.

## Introduction

The Health Behaviour in School-aged Children (HBSC) study is an international research collaboration conducted with the World Health Organization that monitors young people’s health and social conditions in nationally representative samples every 4 years (Cosma et al., 2020; Currie and Morgan, 2020; Eriksson et al., 2019). Analyses based on problematic social media use (HBSC; van den Eijnden et al., 2016) and related surveys consistently show that adolescents reporting high PSMU also report a wide range of difficulties, including sleep problems, poorer physical health, obesity and substance use (Boniel-Nissim et al., 2022; Buda et al., 2021; Khan et al., 2024; Oduro et al., 2023; Zhang et al., 2022), bullying and cyberbullying involvement (Craig et al., 2020; Marengo et al., 2021), and lower family support, peer support, school support, and well-being (Blinka et al., 2020; Boer et al., 2020; Boniel-Nissim et al., 2022; Faltynkova et al., 2020; Lahti et al., 2024). However, these findings are derived from variable-centered associations and therefore indicate co-variation at the population level rather than whether such problems are concentrated within the same individuals. The observed correlations may reflect a subgroup of adolescents with broad psychosocial maladjustment, but they may also arise because adolescents experience different constellations of difficulties. Therefore, the interpretation of a high PSMU score in monitoring data remains uncertain.

PSMU is repeatedly associated with measures typically used as stress-response indicators. Both addiction-oriented and compensatory use frameworks assume that problematic engagement with social media is related to difficulties in managing demands, emotions, or negative life experiences (Brand et al., 2016; Brand et al., 2019; Kardefelt-Winther, 2014). Empirically, PSMU is consistently associated with perceived stress (Shannon et al., 2022; van den Eijnden et al., 2016) and with stress-related reactions such as psychosomatic complaints (Boniel-Nissim et al., 2022; Marino et al., 2020; Peprah et al., 2024; van Duin et al., 2021). Perceived stress and psychosomatic complaints are themselves closely linked (Corell et al., 2022; Låftman and Östberg, 2024), forming part of a well-established stress–response system in adolescence.

Perceived stress and psychosomatic complaints were selected here because they represent complementary components of the adolescent stress-response system. Perceived stress reflects the cognitive appraisal that environmental demands exceed coping resources, whereas psychosomatic complaints represent affective and physiological manifestations of sustained strain. Together, they provide an established indicator of underlying distress in adolescent population research and therefore allow evaluation of whether high PSMU occurs within a broader stress-related vulnerability context rather than as an isolated behavior.

Taken together, this literature suggests that PSMU may not represent an isolated behavioral problem but could function as an indicator of vulnerability. However, existing findings are almost entirely based on variable-centered associations. Such associations demonstrate population-level co-variation but do not reveal whether elevated PSMU, stress, and psychosomatic complaints cluster within the same individuals in distinct psychosocial configurations. Consequently, when an adolescent reports a high PSMU score in population monitoring data, the meaning of that score remains uncertain: it may identify adolescents experiencing broader psychosocial difficulties, or it may reflect heterogeneous groups with different levels of adjustment. Person-centered analyses can test this because variable-centered analyses do not address individual-level classification.

The present study examined how PSMU clusters with perceived stress and psychosomatic complaints in nationally representative adolescent samples. Using HBSC data collected in 2022 from five Nordic countries (Denmark, Finland, Iceland, Norway, and Sweden), the study examines whether adolescents with high PSMU predominantly occur in high-stress/high-symptom profiles.

The present study evaluates the meaning of PSMU as a surveillance indicator. A key challenge for behavioral surveillance indicators is context dependency. The HBSC multi-country design allows testing whether the indicator functions similarly across national settings. We used multiple independent national samples, five Nordic countries, to test whether the meaning of a high PSMU score is stable across populations rather than being an artifact of a single social context.

Accordingly, the central issue is not whether PSMU shows statistical associations with psychosocial difficulties at the population level, which has been repeatedly demonstrated, but whether a high individual PSMU score consistently identifies adolescents experiencing broader psychosocial problems. The present study therefore evaluates the individual-level meaning of a high PSMU score rather than the strength of associations between variables.

### Present study

The interpretation of PSMU in adolescent research remains unclear. Much of the literature implicitly treats PSMU as an indicator of psychosocial problems, assuming that adolescents who report high levels of problematic use also experience broader emotional and social difficulties. However, variable-centered associations cannot determine whether PSMU consistently marks a general risk condition or whether high PSMU is observed among adolescents with markedly different levels of psychosocial adjustment.

To address this issue, the present study adopts a person-centered approach. The study evaluates the interpretive validity of PSMU as a population surveillance indicator rather than testing causal models of social media effects on mental health. We first identified profiles based on the joint distribution of PSMU, perceived stress, and psychosomatic complaints—three indicators central to the adolescent stress-response system. We then examined whether these profiles differed systematically in external psychosocial conditions, including cyberbullying involvement, relational functioning, school experiences, loneliness, and well-being. If PSMU functions as a general marker of psychosocial problems, adolescents with high PSMU should predominantly appear in profiles characterized by elevated stress and broader psychosocial adversity.

Using data from the 2022 Health Behaviour in School-aged Children (HBSC) survey in the Nordic countries, we addressed two questions. First, do distinct profiles emerge based on the joint distribution of PSMU, perceived stress, and psychosomatic complaints? Second, do adolescents characterized by high levels on PSMU show the most adverse psychosocial conditions in family, peer, school, and personal domains?

The aim of the present study was to examine the status of problematic social media use (PSMU) within adolescent psychosocial functioning. Specifically, we investigated whether PSMU reflects a distinct behavioral condition or whether it primarily measures broader psychosocial distress. Using cross-sectional data, we evaluated how the variance associated with PSMU overlaps with mental health problems and everyday psychosocial adversities, interpreting these associations as evidence regarding the construct validity of PSMU rather than as indicators of causal direction.

Cluster analysis provides a data-driven approach to identifying subgroups characterized by distinct constellations of risk indicators across countries. We test whether clusters based on psychosomatic complaints, perceived stress, and PSMU identify adolescents with broader psychosocial maladjustment. Maladjustment is defined using external validation variables: cyberbullying involvement, negative interpersonal and school experiences, and problematic personal conditions, all of which have previously been linked to PSMU (Blinka et al., 2020; Boer et al., 2020; Boniel-Nissim et al., 2022; Lahti et al., 2024).

## Methods

### Study population

The total cross-sectional sample comprised 7,202 adolescents (with complete data for the clustering variables): Denmark (812 boys and 830 girls), Finland (472 boys and 509 girls), Iceland (1,465 boys and 1,392 girls), Norway (497 boys and 477 girls), and Sweden (712 boys and 671 girls). The mean age ranged from 15.75 to 15.89 years across countries. The effect size comparing country differences for age (η^2^ = 0.01) was negligible. Participants were selected by random cluster sampling within strata defined by geographical area to ensure nationally representative datasets. School classes served as sampling units and stratification was proportional. In Iceland all schools were invited to participate. To achieve the target number of participants, non-participating schools were replaced by other schools belonging to the same stratum, which is defined by school size or other relevant aspects.

Data were collected using self-administered, internationally standardized questionnaires during school hours following teacher instruction. The study was conducted through online questionnaires in all countries except for a minority of participants in Sweden who answered a paper-and-pencil questionnaire. The data collection methodology is described in the HBSC study protocol 2021/22 (Inchley et al., 2022), which ensures consistency in sampling methods, survey instruments, and data collection procedures.

All procedures complied with the Declaration of Helsinki and were reviewed by relevant national ethical and data protection authorities. Formal ethical approval was not required in accordance with national legislation and institutional requirements. All participants received written and verbal information about the study, including that participation was voluntary, and informed assent was obtained from the adolescents themselves.

### Measures

Three variables were used for clustering: psychosomatic complaints, perceived stress, and PSMU. In addition, four sets of external variables were included to evaluate the external validity and interpretability of the cluster solution: (a) online communication with friends, (b) cyberbullying (victimization and perpetration), (c) negative interpersonal and school experiences (difficulty communicating with parents, lack of peer support, negative school experiences), and (d) problematic personal conditions (loneliness and low well-being). Cluster and comparison variables were also examined in relation to sex and socioeconomic status (SES). All measures are described in Table 1.

**Table 1:** Measures used in the study.

Measures	Items and alpha reliability	Response scale
Psychosomatic complaints (HBSC-SCL) is an eight-item measure of individuals’ affective and physiological symptoms, asking the adolescents: “In the last 6 months, how often have you had the following.” and the eight items: ‘headache’, ‘stomachache’, ‘backache’, ‘feeling low’, ‘irritability or bad temper’, ‘feeling nervous’, ‘difficulty falling asleep’ and ‘feeling dizzy’. A factor analysis across countries revealed one factor with factor scores ranging from 0.59 to 0.79 (average 0.71).	8/0.87	1 (rarely or never) to 5 (about every day)
Problematic social media use was assessed using the 9-item Social Media Disorder Scale developed by van den Eijnden et al. (2016). The scale has demonstrated good psychometric properties and has been shown to be reliable for cross-national comparisons (Boer et al., 2022). Following the instruction “During the past year, have you…,” the respondents answered items like “regularly found that you cannot think of anything else but the moment that you will be able to use social media again?.”	9/0.82	1 (no) and 2 (yes)
Perceived stress is a 2-item measure based on Cohen’s Perceived Stress Scale (PSS-10) measuring individuals’ cognitive appraisal of lacking control: “How often have you “Felt unable to control important things in life,” and “Felt difficulties were piling up so high that you could not overcome them.” The correlation between the two items was 0.52 (*p* < 0.001).	2/ -	1 (never) to 5 (very often)
Online communication with friends. After a short definition on online communication, the respondents were asked how often they had online contacts with closed friend(s), and friends from a larger friend group. An option that the questions did not apply was assigned a missing value (about 6%). The correlation between the two questions was 0.62 (*p* < 0.001).	2/ -	1 (never or almost never) to 5 (almost all the time throughout the day).
Being cyberbullied: After a written definition of cyberbullying, the respondents were asked how often they had taken part in cyberbullying others. Cyberbullying others: Similarly, for being exposed to bullying, after introducing a definition of being cyberbullied, the respondents were asked how often had been cyberbullied.	2/ -	1 (I have not cyberbullied another person in the past couple of months) to 5 (several times a week) 1 (I have not been cyberbullied in the past couple of months) to 5 (several times a week)
Difficulties communication with mothers and fathers was measured by asking adolescents how easy it was for them to talk to their father and mother, respectively, about things that really bothered them. Adolescents who answered that they had no contact with the specific parent were assigned a missing value. The correlation between the perceived ease of communicating with fathers and mothers was 0.62 (*p* < 0.001).	2/ -	1 (very easy) to 4 (very difficult)
Lack of peer support was a 4-item scale (from the Multidimensional Scale of Perceived Social Support by Zimet et al., 1988) “Friends try to help,” “Can count on friends,” “Friends to share joys with,” and “Can talk about problems with friends.”	4/0.93	1 (very strongly disagree) to 7 (very strongly agree), reverse coded
Negative school experiences was a 2-item measures that combined perceived school pressure and not liking school. The first item asked about present school pressures. The second item asked about the respondents liking school at present. The correlation between the two items was low, 0.25, but both differentiated substantially between the cluster groups in a similar way.	2/ -	1 (not at all) to 4 (a lot) 1 (like a lot) to 4 (not at all)
Feeling lonely was a single item “During the past 12 months, how often have you felt lonely?.”	1/ -	1 (never) to 5 (always)
Low well-being, in the internal protocol of HBSC labeled WHO-5 Well-being Index, consists of five items asking the respondents, how often during the last 2 weeks “I have felt cheerful and in good spirits”, “I have felt calm and relaxed,” “I have felt active and vigorous,”” I woke up feeling fresh and rested,” and “My daily life has been filled with things that interest me.” Reversely coded.	5/0.87	1 (at no time) to 5 (all of the time)
Perceptions of the family’s socioeconomic conditions was a single item: “How well off do you think your family is?.” This measure was not collected in Denmark.	1/ -	1 (very well off) to 5 (not at all well off), reverse coded
Sex	1/ -	0 (boys) and 1 (girls)

The measures employed in the present study (PSMU, psychosomatic complaints, perceived stress, psychosocial indicators) are part of the core or optional HBSC item packages and have been evaluated in previous international methodological studies. Documentation of sampling, measurement development, and psychometric testing is available in the HBSC international protocol and methodological reports (Inchley et al., 2022).

### Statistical analyses

Cluster analysis was conducted using the three standardized clustering variables. To determine the appropriate number of clusters, hierarchical cluster analysis with Ward’s method was first performed. We applied Bergman and colleagues’ screening criterion for cluster structure that a cluster solution should explain at least 67% of the total error sums of squares (Bergman et al., 2003). Once the number of clusters was established, K-means clustering was used to obtain the final solution, following recommendations by Kinder et al. (1991). Cluster centroids were interpreted using the following guidelines: values < −0.70 were considered low, values between −0.70 and 0.70 average, and values > 0.70 high.

Effect sizes were evaluated using Cramér’s V (0.02 small, 0.20 medium, ≥0.60 large) and eta^2^ (0.01 small, 0.06 medium, ≥0.14 large).

## Results

We first examined whether online communication with friends and PSMU varied across the Nordic countries. In both cases, the effect size was negligible (η^2^ = 0.00), indicating virtually no between-country differences.

As expected, psychosomatic complaints were moderately correlated with perceived stress (*r* = 0.49) and PSMU (*r* = 0.29), and PSMU was correlated with stress (*r* = 0.27). Bivariate correlations between PSMU and the seven psychosocial comparison measures (cyberbullying, negative interpersonal and school experiences, and negative well-being) were all statistically significant (*p* < 0.001) but generally small (*r* = 0.19). Correlations ranged from 0.08 (classmate support) to 0.25 (loneliness). When controlling for psychosomatic complaints and perceived stress, five of these correlations were reduced to approximately 0.05, while associations with cyberbullying victimization (0.17) and perpetration (0.16) remained somewhat stronger.

Overall, these findings indicate that PSMU shows weak associations with psychosocial conditions at the population level, underscoring the need for person-centered analyses to identify vulnerable subgroups. This pattern suggests that reliance on PSMU alone as a screening indicator may lead to substantial misclassification of adolescents’ psychosocial risk.

The correlation between online communication with friends and psychosomatic complaints was 0.02 and non-significant. Bivariate correlations between online communication and the seven psychosocial comparison measures were, in most cases, negative and very small (approximately −0.08), with a more pronounced negative association for poor classmate support (*r* = −0.22). Compared with PSMU, a higher frequency of online communication with friends was thus associated with slightly more favorable psychosocial conditions, particularly greater perceived support from classmates.

### Cluster solutions

We used Bergman’s 67% screening guideline for checking the cluster structure (Bergman et al., 2003). An eight-cluster solution explained 68.6% of the error sums of squares. However, two of these eight clusters produced redundant profile splits and no new profiles. First, in the eight-cluster solution, one cluster was divided into two profiles that differed only in degree: one displayed high values on a focal variable, the other very high values, while the remaining two variables were of comparable magnitude across both profiles. Second, another cluster was similarly split into two subclusters that showed equivalent patterns on the external validating measures. Because cluster solutions in applied health research should balance statistical fit with interpretability and parsimony (Hair et al., 2018), we selected the six-cluster solution (62.3%), which yielded distinct and theoretically meaningful profiles.

Country-specific analyses (Supplementary Table 1) indicated a largely comparable pattern across the five Nordic countries, although individual profiles were occasionally absent. Overall, similarity in 28 of the 30 possible profile–country combinations were observed. Deviations mainly reflected milder variants of the complaints profile (elevated rather than high complaints) and the absence of a clear multi-problem group in Finland. Thus, between-country variation concerned the prevalence and intensity of the profiles rather than their configuration, supporting analysis of a shared Nordic typology. Accordingly, the pooled solution can be interpreted as describing comparable types of adolescents in each country rather than reflecting a pattern driven by a single national sample.

### Description of the six clusters

The six-cluster solution is presented in Table 2. A “Multi-problem” cluster (7.5%) showed high levels of psychosomatic complaints, stress, and PSMU. A second cluster (“High psychosomatic complaints and stress”, 13.5%) showed similarly high psychosomatic complaints and stress but average levels of PSMU. A third cluster (“PSMU exclusively”, 12.7%) showed high PSMU without elevated psychosomatic complaints or stress. A fourth cluster (“High complaints exclusively”, 17.8%) showed elevated psychosomatic complaints only. Two additional clusters reflected low (27.1%) or average (21.4%) levels across all three indicators.

**Table 2:** Cluster profiles based on psychosomatic complaints, perceived stress, and problematic social media use (PSMU) and their associations with sex, online communication, cyberbullying, interpersonal and school experiences, well-being, and socioeconomic status.

Clustering variables	Cluster profiles					
	Low complaints and stress	Average levels	High complaints exclusively	High complaints and stress	PSMU exclusively	Multi-problem
Psychosomatic complaints	−0.84	−0.56	0.86	1.19	−0.32	1.05
Perceived stress	−1.09	0.39	−0.12	1.38	−0.10	0.81
PSMU	−0.62	−0.53	−0.25	0.06	1.19	2.21
*N*	1957	1,543	1,280	969	913	540
%	27.1	21.4	17.8	13.5	12.7	7.5
% boys^a^	38.1^h^	27.6^h^	11.5^l^	5.4^l^	12.5	4.8^l^
% girls	17.3^l^	15.9^l^	23.5^h^	20.6^h^	13.1	9.6^h^
Comparison measures						
Online contacts						
Online communication with friends^1^	−0.01^b^	−0.11^a^	−0.02^b^	−0.01^b^	0.04^b^	0.11^b^
Cyberbullying						
Been cyberbullied^2^	−0.22^e^	−0.13^d^	−0.02^c^	0.14^b^	0.08^b^	0.63^a^
Cyberbullied others^3^	−0.15^d^	−0.08^c^	−0.02^c^	−0.01^c^	0.10^b^	0.50^a^
Negative interpersonal and school experiences						
Difficult to talk to parents^4^	−0.41^f^	−0.12^e^	0.20^c^	0.57^a^	0.02^d^	0.46^b^
Lack of peer support^5^	−0.25^d^	−0.03^c^	0.09^b^	0.30^a^	−0.02^c^	0.23^a^
Negative school experiences^6^	−0.58^e^	−0.13^d^	0.25^c^	0.76^a^	−0.08^d^	0.66^b^
Negative well-being						
Feeling lonely^7^	−0.62^f^	−0.15^e^	0.29^c^	0.81^a^	0.01^d^	0.72^b^
Low well-being^8^	−0.64^e^	−0.18^d^	0.34^c^	0.92^a^	−0.11^d^	0.67^b^
Low SES						
Family is not well off^9^	−0.24^d^	−0.05^c^	0.09^b^	0.28^a^	−0.03^c^	0.28^a^

Values represent standardized scores (Z-scores) for the clustering variables; higher values indicate higher levels of the respective indicator. Percentages of boys and girls indicate the distribution of sex within each cluster. Differences between clusters in comparison measures were tested using one-way ANOVA. Different superscript letters (b–f) indicate statistically significant differences between cluster means within each row (*p* < 0.05). The total sample size in the cluster analysis was *N* = 7,202. Adjusted standardized residuals from chi-square analyses are indicated by superscripts (h = higher than expected; l = lower than expected).

a χ^2^ (df = 5, *N* = 7,045) = 899.21, *p* < 0.001, Cramer’s V = 0.36.

1 F(5, 6,358) = 6.27, *p* < 0.001, η^2^ = 0.00.

2 F(5, 7,159) = 79.21, *p* < 0.001, η^2^ = 0.05.

3 F(5, 7,155) = 42.43, *p* < 0.001, η^2^ = 0.03.

4 F(5, 7,116) = 183.49, *p* < 0.001, η^2^ = 0.11.

5 F(5, 7,164) = 51.58, *p* < 0.001, η^2^ = 0.03.

6 F(5, 7,195) = 397.77, *p* < 0.001, η^2^ = 0.22.

7 F(5, 7,183) = 490.89, *p* < 0.001, η^2^ = 0.25.

8 F(5, 7,191) = 246.93, *p* < 0.001, η^2^ = 0.15.

9 F(5, 5,633) = 40.87, *p* < 0.001, η^2^ = 0.04.

Sex differences were pronounced. Boys were overrepresented in the two favorable clusters (“Low complaints and stress” and “Average levels”), whereas girls were overrepresented in all clusters characterized by elevated psychosomatic complaints. No sex differences were observed in the “PSMU exclusively” cluster.

The key observation is that adolescents with high PSMU were not concentrated in a single high-risk group. Instead, high PSMU appeared both within a multi-problem profile characterized by elevated stress and psychosomatic complaints and within a profile showing otherwise typical psychosocial functioning. Thus, identical PSMU scores occurred in adolescents with markedly different levels of psychosocial adjustment.

### External correlates of the cluster profiles

Online communication with friends showed only minor differences across cluster groups (Table 2). Cyberbullying victimization and perpetration were most prevalent in the “Multi-problem” cluster. Adolescents in both the “Multi-problem” and “High complaints and stress” clusters reported the highest levels of loneliness and the lowest levels of well-being, with moderate to large effect sizes. Notably, adolescents with high psychosomatic complaints and stress but average PSMU showed psychosocial difficulties comparable to—or greater than—those in the “Multi-problem” cluster, except for cyberbullying involvement.

In contrast, adolescents in the “PSMU exclusively” cluster showed only slightly elevated cyberbullying scores and otherwise reported average interpersonal, school, and well-being outcomes. Thus, high PSMU without co-occurring distress indicators does not correspond to broad psychosocial difficulties. At the same time, the absence of co-occurring distress does not imply that elevated PSMU reflects a beneficial condition, and future research should examine whether adolescents high in PSMU differ in strengths-based or adaptive outcomes not captured by the present indicators. It should also be emphasized that adolescents high in complaints and stress but average PSMU were as impaired (or more) than the multi-problem group with high PSMU. This indicates that PSMU does not uniquely identify adolescents with the most pronounced psychosocial difficulties.

Finally, some adolescents may have complete data for only a subset of the clustering variables and could therefore differ systematically. Thus, we compared adolescents with complete clustering data (*n* = 7,202) to those with partial responses (*n* = 737) using t-tests across the nine validation measures. Adolescents included in the cluster analysis differed only marginally from those excluded because of incomplete responses. Across the psychosocial validation measures, group differences were generally negligible. Involvement in bullying, both victimization and perpetration, was somewhat more common among adolescents with incomplete responses, but the effects were small (Cohen’s *d* ≈ 0.22). Overall, the findings point to limited selective nonresponse rather than systematic exclusion of adolescents with elevated psychosocial problems.

## Discussion

A substantial body of research has documented associations between problematic social media use (PSMU) and adolescent mental health indicators, including depressive symptoms, anxiety, psychosomatic complaints, and reduced well-being (Boer et al., 2020; Huang, 2022; Keles et al., 2020; Pezzi et al., 2024). The present study addressed the interpretive meaning of a commonly used measure of problematic social media use. Rather than examining whether PSMU correlates with indicators of mental health, the analyses evaluated what information a high score conveys about psychosocial functioning. Across the Nordic samples, high PSMU scores occurred in multiple psychosocial contexts. Adolescents with similarly elevated scores appeared in qualitatively different profiles, including a multi-problem configuration characterized by high stress and psychosomatic complaints and a profile showing otherwise typical functioning.

A high score occurred within broader distress but also appeared in adolescents without elevated stress or psychosomatic symptoms. Consequently, the psychological meaning of PSMU depends on the context in which it occurs. The results therefore clarify the construct validity of PSMU in population research: high scores identify heterogeneous groups rather than a coherent risk condition.

A screening indicator is informative when individuals scoring high on the indicator are concentrated among those with the target condition. In the present study, adolescents with high PSMU were distributed across markedly different psychosocial profiles, and adolescents with the most adverse psychosocial conditions were frequently identified without elevated PSMU. It means that the latent organization of psychosocial distress in survey data is not centered on the PSMU indicator.

The present findings also clarify how the term “problematic” should be understood. Elevated PSMU did not uniformly co-occur with broad psychosocial adversity. Thus, high PSMU scores cannot be taken to indicate generalized maladjustment, nor should they be equated with high but normative digital engagement. The construct captures self-reported difficulties in regulating social media use, but these difficulties appear in heterogeneous psychosocial contexts. In this respect, PSMU reflects perceived dysregulation rather than a clinical condition, and its meaning depends on the broader profile within which it occurs.

### Stability of cluster patterns across countries

The multi-country design allowed us to examine whether the meaning of a high PSMU score depended on national context. Each national HBSC sample constituted an independent population studied with identical methods but embedded in somewhat different school, peer, and digital environments. The similar profile structure across countries showed that high PSMU appeared both within a multi-problem configuration and among adolescents with otherwise typical functioning in every sample. This consistency indicates that the observed heterogeneity is not specific to a single national context. Accordingly, in population monitoring the interpretation of a high PSMU score is unlikely to change simply because the survey is conducted in another national setting. It appears as the meaning of the indicator show a consistent interpretation across populations. However, because all samples were drawn from Nordic welfare contexts with relatively similar digital infrastructures and policy environments, replication in non-Nordic settings is needed before broader international generalizations can be made.

### Heterogeneity of PSMU

The multi-problem cluster displayed an adverse psychosocial profile, including strained relationships with parents and peers, negative school experiences, loneliness, and low well-being. This finding identifies adolescents with the most adverse concurrent psychosocial profiles. The prevalence estimates showed that PSMU co-occurred with elevated psychosomatic complaints and stress in approximately 8% of adolescents. In contrast, a larger proportion (14%) exhibited elevated psychosomatic complaints and stress but not high PSMU. In effect, the largest distressed group does not include high PSMU.

### PSMU and time-based indicators

Using large-scale databases, researchers have reached markedly different conclusions regarding the role of social media and general internet use in the rise of adolescents’ mental health problems over the past decade and a half. Some have argued that online social media has exerted substantial direct or indirect effects on increasing mental health problems (Twenge et al., 2018), whereas others contend that its practical contribution is minimal (Orben and Przybylski, 2019), emphasizing cross-sectional heterogeneity in internet and mobile use and unclear causal directions (Odgers and Jensen, 2020; Orben, 2020). Importantly, these divergent conclusions were largely based on analyses of time spent online—an indicator that, in the present study, did not differentiate between the identified cluster groups. These studies have likewise reported very small cross-sectional associations, consistent with our finding of a non-significant correlation between frequency of online communication with friends and psychosomatic complaints (*r* = 0.02). Consistent with this, intensity of online communication with friends also failed to distinguish high-risk profiles: adolescents who communicated frequently online were not more likely to belong to the high-PSMU or multi-problem clusters.

PSMU represents a more refined indicator of problematic online engagement than simple frequency of communication with friends and was more substantially related to psychosomatic complaints (*r* = 0.29). Whereas frequency of online communication with friends was slightly negatively associated with psychosocial adversities (mean *r* ≈ −0.08), PSMU showed positive associations (mean *r* ≈ 0.19). Taken together, these findings suggest that time-based measures of internet use are too crude to capture psychosocial vulnerability. It is not the quantity of online interaction per se that differentiates adolescents at risk, but the qualitative features reflected in problematic or dysregulated use. At the same time, the cluster results demonstrate substantial heterogeneity: some adolescents high in PSMU showed no signs of psychosocial adversity, whereas others with similarly high PSMU reported markedly elevated problems.

The observed heterogeneity is developmentally plausible. Adolescence is characterized by marked variability in peer orientation, emotion regulation, and social sensitivity. Thus, a behavioral indicator such as PSMU is unlikely to map uniformly onto psychosocial risk across individuals. From a surveillance perspective, this underscores that PSMU should not be interpreted as a stand-alone risk marker, but in relation to adolescents’ broader psychosocial context.

Notably, the person-centered findings are consistent with variable-centered research showing weak average associations between social media use and maladjustment, alongside substantial heterogeneity across adolescents. Although the analytic strategies differ, both approaches converge in indicating that aggregate effects are small and that associations depend on underlying configurations of risk and context.

### Relation to theory

Both dysfunction- and motivation-oriented models posit that adolescents may turn to social media in response to stress or adverse psychosocial experiences, with problematic use developing when regulation fails or coping attempts are ineffective (Brand et al., 2019; Kardefelt-Winther, 2014; Wegmann and Brand, 2016). Our design evaluates what information a survey score carries relative to what theories implicitly assume that score represents. The study, conducted as a screening-indicator evaluation study embedded in a large epidemiological monitoring system (HBSC), attempted to understand if a high PSMU score functions as a marker of broad psychosocial problems. We find that stress indicators (perceived stress combined with psychosomatic complaints) correspond more strongly to broad psychosocial difficulties. In effect, the interpretation of the PSMU score depends on its co-occurring symptom context.

The role of cyberbullying involvement helps specify when PSMU is embedded in broader maladjustment. Cyberbullying was concentrated in the multi-problem cluster and was largely absent in the high-PSMU-only cluster. One plausible interpretation is that the association between high PSMU and psychosocial adversity observed in variable-centered studies may partly reflect the subgroup of adolescents whose problematic use occurs within a broader distress context that includes cyberbullying involvement. Cyberbullying was rare among adolescents with psychosocial distress but average PSMU, and equally rare among adolescents with high PSMU but otherwise typical psychosocial functioning. Therefore, cyberbullying was not elevated in the PSMU-only cluster, suggesting that it does not uniformly accompany high PSMU. The findings indicate two contexts in which the same score appears: one embedded in broader psychosocial maladjustment and one occurring among otherwise well-adjusted adolescents. The concentration of cyberbullying involvement in the multi-problem profile is consistent with prior research showing that hostile online interactions are closely linked to adolescent maladjustment (Craig et al., 2020; Kowalski et al., 2014; Modecki et al., 2014; Tokunaga, 2010). Taken together, the findings suggest that a high PSMU score does not consistently signal psychosocial risk: cyberbullying appears primarily when high PSMU occurs within a broader maladjustment profile.

### Sex differences

Consistent with previous research, boys were overrepresented in clusters indicating few or no problems, whereas girls were overrepresented in clusters characterized by elevated psychosomatic complaints (Cosma et al., 2020; Stattin and Eriksson, 2024). This pattern underscores the central role of psychosomatic symptoms as an indicator of adolescent mental health problems in Nordic countries.

Sex differences also help clarify heterogeneity among adolescents with high PSMU. Although girls predominated in the multi-problem cluster, girls and boys were represented in similar proportions in the PSMU-only cluster. To our knowledge, this difference in sex distribution across PSMU profiles has not previously been reported. When PSMU co-occurred with psychosocial distress and stress, girls were overrepresented; when PSMU occurred without such difficulties, the sex difference largely disappeared. Thus, the commonly observed female preponderance in PSMU appears to reflect its co-occurrence with psychosocial distress rather than PSMU itself.

### Practice implications

The findings argue against one-size-fits-all prevention strategies and suggest that screening based solely on problematic social media behavior is likely to misidentify adolescents’ needs. The adolescents with the most adverse profiles were characterized by co-occurring stress-related symptoms and difficulties in their everyday social environments, including strained parent communication, low peer support, and negative school experiences. By contrast, adolescents with elevated PSMU in the absence of these conditions showed largely typical psychosocial functioning. Therefore, from a public-health monitoring perspective, psychosomatic complaints, perceived stress, and relational functioning may provide a more informative basis for identifying adolescents requiring support than PSMU in isolation.

### Missing data analyses

The missing data analyses indicate that the clustering solution was not driven by listwise selection. Adolescents excluded because of incomplete responses showed no meaningful differences on most psychosocial indicators, and the differences that appeared were small. The somewhat higher involvement in bullying among excluded adolescents likely reflects response patterns in more socially vulnerable groups rather than absence of the clustering constructs themselves. Accordingly, the cluster profiles are unlikely to represent artifacts of selective responding, although the prevalence of the most vulnerable cluster may be slightly underestimated.

### Strengths and limitations

Strengths of the study include the ability to interpret a surveillance indicator through external validation across quasi-replications (large, nationally representative samples, five Nordic countries, harmonized HBSC protocol) and a person-centered analytic approach that captures heterogeneity in adolescent health profiles. This approach is particularly well suited for public-health surveillance.

A central limitation is that we cannot determine whether PSMU is an antecedent, a consequence, or a parallel expression of distress. Limitations also include person-centered classification uncertainty (profiles are descriptive groupings, not natural types), common method variance due to self-report, negative affectivity or response style bias, and a cross-sectional design. A related concern in PSMU research is potential construct contamination. Because some PSMU items refer to loss of control or conflict, associations with maladjustment may partly reflect shared distress content or generalized negative affectivity in self-report data. This limitation applies here as well. However, the observed heterogeneity in cluster profiles complicates a pure contamination interpretation: elevated PSMU did not uniformly co-occur with elevated psychosocial adversity. Thus, shared method variance alone is unlikely to explain the pattern of findings, although longitudinal and multi-informant studies would help further clarify this issue.

Notably, the mean age of the sample approximates the 16-year threshold currently debated or implemented in some policy contexts concerning social media access. Although the present findings do not speak directly to regulatory decisions, they provide contextually timely evidence regarding the heterogeneity of elevated PSMU in mid-adolescence.

## Conclusion

High engagement or self-reported loss-of-control experiences on social media, by themselves, provided limited information about adolescents’ mental health. Instead, the psychological relevance of PSMU depended on the context in which it occurred. The association between PSMU and broader psychosocial difficulties was observed within the multi-problem profile rather than within the PSMU-only profile. When these conditions were absent, high PSMU was not accompanied by a broad pattern of psychosocial problems.

Overall, the findings indicate that a high PSMU score in population surveys should be interpreted cautiously when used as a stand-alone indicator. Instead, it appears in heterogeneous psychosocial contexts, including both adolescents with broader distress and adolescents with otherwise typical functioning. Interpretation of high scores therefore requires consideration of accompanying stress-related symptoms and social context. What does a high PSMU score mean in population monitoring? The answer seems to be that it is a non-specific behavioral signal whose interpretation requires accompanying stress indicators.

## Data Availability

The data presented in this study are available on request from the Data Management Center: http://www.hbsc.org/data/ at the Department of Health Promotion and Development at the University of Bergen, Norway. Further inquiries can be directed to the corresponding author.
