# Supplementary material for: Problematic social media use and psychosocial conditions
Source: Front Psychol. 2026 Mar 27;17:1778148. doi: 10.3389/fpsyg.2026.1778148 (PMC13067289; doi:10.3389/fpsyg.2026.1778148)
Supplement: Supplementary file 1 [file Supplementary_file_1.docx]

**Supplementary Table 1.** Cluster analyses of psychosomatic complaints, perceived stress, and problematic social media use (PSMU) in each of the five Nordic countries.

_________________________________________________________________________________________________________________

Low complaints Average High High complaints Problematic Multi-

and stress levels complaints and stress social media use problem

exclusively exclusively

_________________________________________________________________________________________________________________

Denmark:

Psychosomatic complaints -0.74 -0.52 0.94 1.53 -0.33 0.99

Perceived stress -0.99 0.48 0.06 1.73 -0.33 0.75

PSMU -0.62 -0.42 -0.34 0.23 1.21 1.41

*N* 512 337 207 140 166 186

% 33.1 21.8 13.4 9.0 10.7 12.1

_________________________________________________________________________________________________________________

Low complaints Average High High complaints Problematic *High complaints*

and stress levels complaints and stress social media use *and problematic*

exclusively exclusively *social media use*

_________________________________________________________________________________________________________________

Finland:

Psychosomatic complaints -0.81 -0.67 0.77 0.96 -0.61 *0.99*

Perceived stress -1.15 0.33 -0.01 1.38 -0.36 *0.38*

PSMU -0.70 -0.59 -0.30 0.13 1.10 *2.00*

*N* 195 177 171 138 103 81

% 22.5 20.5 19.8 16.0 11.9 9.4

_________________________________________________________________________________________________________________

Low complaints Average Elevated levels High complaints Problematic Multi-

and stress levels of complaints and stress social media problem

exclusively use exclusively

_________________________________________________________________________________________________________________

Iceland:

Psychosomatic complaints -0.90 -0.52 0.64 1.32 -0.46 1.04

Perceived stress -1.05 0.48 -0.15 1.33 -0.18 0.86

PSMU -0.62 -0.51 -0.26 -0.15 1.45 1.65

*N* 739 472 520 324 267 267

% 28.5 18.2 20.1 12.5 10.3 10.3

_________________________________________________________________________________________________________________

Low complaints Average *Average* High complaints Problematic Multi-

and stress levels *levels* and stress social media use problem

exclusively

_________________________________________________________________________________________________________________

Norway:

Psychosomatic complaints -0.85 -0.39 *0.44* 1.44 -0.19 1.40

Perceived stress -0.88 0.55 *-0.25* 1.20 -0.03 1.13

PSMU -0.64 0.15 *-0.34* -0.15 2.35 1.55

*N* 285 171 179 104 50 75

% 33.0 19.8 20.7 12.0 5.8 8.7

_________________________________________________________________________________________________________________

Low complaints Average Elevated levels High complaints Problematic Multi-

and stress levels of complaints and stress social media use problem

exclusively exclusively

_________________________________________________________________________________________________________________

Sweden:

Psychosomatic complaints -0.87 -0.67 0.66 1.15 -0.08 1.08

Perceived stress -1.06 0.62 -0.32 1.13 0.03 0.83

PSMU -0.60 -0.54 -0.32 0.00 1.20 2.37

*N* 362 241 265 201 168 99

% 27.1 18.0 19.8 15.0 12.6 7.4

__________________________________________________________________________________________________________________

*Note*. The clusters in the separate countries which differ from the clusters combining all countries are italicized.

The following guidelines were used for interpreting the clusters: a low standardized centroid value is < -0.70, an average value is between -0.70 and 0.70, and a high value is > 0.70.

The cluster analyses for Denmark and Iceland initially produced two clusters of "Problematic social media use exclusively". One cluster had high scores for problematic social media use and the other cluster had even higher scores for the same clustering variable. The levels of the other clustering variables were about the same. To avoid the separation into two clusters, the standardized scores for problematic social media use were recoded prior to the cluster analyses in these countries so that all scores above 2.5 were recoded to 2.
